# Novel methods for elucidating modality importance in multimodal electrophysiology classifiers

**DOI:** 10.3389/fninf.2023.1123376

**Published:** 2023-03-15

**Authors:** Charles A. Ellis, Mohammad S. E. Sendi, Rongen Zhang, Darwin A. Carbajal, May D. Wang, Robyn L. Miller, Vince D. Calhoun

**Affiliations:** ^1^The Wallace H. Coulter Department of Biomedical Engineering, Georgia Institute of Technology, Emory University, Atlanta, GA, United States; ^2^Tri-Institutional Center for Translational Research in Neuroimaging and Data Science, Georgia State University, Georgia Institute of Technology, Emory University, Atlanta, GA, United States; ^3^McLean Hospital and Harvard Medical School, Boston, MA, United States; ^4^Hankamer School of Business, Baylor University, Waco, TX, United States; ^5^The Wallace H. Coulter Department of Biomedical Engineering, Georgia Institute of Technology, Atlanta, GA, United States; ^6^Department of Computer Science, Georgia State University, Atlanta, GA, United States

**Keywords:** multimodal classification, explainable deep learning, sleep stage classification, electrophysiology, electroencephalography, electrooculography, electromyography

## Abstract

**Introduction:**

Multimodal classification is increasingly common in electrophysiology studies. Many studies use deep learning classifiers with raw time-series data, which makes explainability difficult, and has resulted in relatively few studies applying explainability methods. This is concerning because explainability is vital to the development and implementation of clinical classifiers. As such, new multimodal explainability methods are needed.

**Methods:**

In this study, we train a convolutional neural network for automated sleep stage classification with electroencephalogram (EEG), electrooculogram, and electromyogram data. We then present a global explainability approach that is uniquely adapted for electrophysiology analysis and compare it to an existing approach. We present the first two local multimodal explainability approaches. We look for subject-level differences in the local explanations that are obscured by global methods and look for relationships between the explanations and clinical and demographic variables in a novel analysis.

**Results:**

We find a high level of agreement between methods. We find that EEG is globally the most important modality for most sleep stages and that subject-level differences in importance arise in local explanations that are not captured in global explanations. We further show that sex, followed by medication and age, had significant effects upon the patterns learned by the classifier.

**Discussion:**

Our novel methods enhance explainability for the growing field of multimodal electrophysiology classification, provide avenues for the advancement of personalized medicine, yield unique insights into the effects of demographic and clinical variables upon classifiers, and help pave the way for the implementation of multimodal electrophysiology clinical classifiers.

## 1. Introduction

Biomedical informatics studies (Lin et al., 2019; Mellem et al., 2020; Zhai et al., 2020), and electrophysiology studies (Niroshana et al., 2019; Phan et al., 2019; Wang et al., 2020; Li et al., 2021) in particular, have increasingly begun to incorporate multimodal data when training machine learning classifiers. Using complementary modalities can enable the extraction of better features and improve classification performance (Wang et al., 2020; Zhai et al., 2020). While multimodal data can improve classifier performance, it can also make explaining models more difficult. This is especially true for state-of-the-art deep learning models. As a result, most studies have not used explainability (Zhang et al., 2011; Kwon et al., 2018; Niroshana et al., 2019; Phan et al., 2019; Wang et al., 2020; Li et al., 2021), which is concerning because transparency is increasingly required to assist with model development and physician decision making (Sullivan and Schweikart, 2019). As such, more multimodal explainability methods need to be developed (Lin et al., 2019; Mellem et al., 2020; Ellis et al., 2021a,b,c,d). In this study, we use automated sleep stage classification as a testbed for the development of multimodal explainability methods. We further present 3 novel approaches that offer significant improvements over existing approaches for use with multimodal electrophysiology data. Specifically, we present a global ablation approach that is uniquely adapted for electrophysiology data. We further present two local methods that can be used to identify personalized electrophysiology biomarkers that would be obscured by global methods. Using the local methods, we perform a novel analysis that illuminates the effects of demographic and clinical variables upon the patterns learned by the classifier.

### 1.1. Automated sleep stage classification as testbed for multimodal explainability

Automated sleep stage classification offers a unique testbed for the development of novel multimodal explainability methods. Automated sleep stage classification has multiple noteworthy characteristics. (1) In practice, clinicians rely on multiple modalities instead of a single modality to manually score sleep stages (Iber et al., 2007). (2) The features differentiating sleep stages and the importance of modalities are well-characterized in a clinical setting (Iber et al., 2007). (3) Multiple large sleep stage datasets are publicly available (Quan et al., 1997; Kemp et al., 2000; Khalighi et al., 2016). (4) A number of studies involving unimodal and multimodal sleep stage classification have been conducted (Rahman et al., 2018; Wang et al., 2020), which could enable data scientists to develop their explainability methods alongside established architectures. Because these characteristics can help us validate our explainability methods and because there is a clinical need for explainability in sleep stage classification, we chose sleep stage classification as a use-case in this study. In the following paragraphs, we briefly review the domain of sleep stage classification and the explainability methods that have been used within the domain, both for unimodal and multimodal classification. A description of sleep stages can be found in the Supplementary section, “Characteristic Features of Sleep Stages.”

### 1.2. Unimodal sleep stage classification and explainability

Typical sleep stage classification approaches involve the classification of 5 stages: Awake, rapid eye movement (REM), non-REM1 (NREM1), NREM2, and NREM3. Many sleep stage classification studies have used unimodal EEG. Some studies have used extracted features for sleep stage classification (Aboalayon et al., 2015; Rojas et al., 2017; Rahman et al., 2018; Michielli et al., 2019), but recent studies have begun to use deep learning methods involving automated feature extraction from raw data (Tsinalis et al., 2016a; Rojas et al., 2017; Supratak et al., 2017; Sors et al., 2018; Mousavi et al., 2019; Eldele et al., 2021). Multiple recent studies have involved explainability methods. In a couple of studies, authors trained convolutional neural networks (CNNs) to classify EEG spectrograms and applied sensitivity or activation maximization (Simonyan et al., 2013) to identify the important features (Vilamala et al., 2017; Ruffini et al., 2019). In other studies, authors trained interpretable machine learning models or deep learning models with layer-wise relevance propagation (LRP) (Bach et al., 2015) to classify power spectral density values and gain insight into the features learned by the classifiers (Chen et al., 2019; Ellis et al., 2021e). A few studies involving deep learning models with raw data have also used explainability methods (Mousavi et al., 2019; Ellis et al., 2021f,g,h). These studies typically seek to identify the spectral features (Nahmias and Kontson, 2020; Barnes et al., 2021; Ellis et al., 2021f,g,h,i) or waveforms (Ellis et al., 2021h,i) learned by neural networks. However, multimodal classification poses unique challenges for explainability that do not exist for unimodal classification.

### 1.3. Multimodal explainability in sleep stage classification and other domains

Most multimodal classification studies, regardless of whether they used extracted features (Phan et al., 2019; Li et al., 2021) or raw data (Niroshana et al., 2019; Wang et al., 2020), have not used explainability methods. Among the few studies involving explainability (Lajnef et al., 2015; Chambon et al., 2018; Pathak et al., 2021), some have used extracted features and forward feature selection (FFS) (Lajnef et al., 2015). Others have used raw data and ablation for insight into modality importance (Pathak et al., 2021). Additionally, some have shown the importance of EEG spectra or performance increases after retraining a model with additional modalities (Chambon et al., 2018). Some multimodal explainability methods are also found in other domains (Lin et al., 2019; Mellem et al., 2020; Porumb et al., 2020). Similar to (Lajnef et al., 2015), one paper used FFS to find key features from clinical scales and imaging features (Mellem et al., 2020). One study used impurity and ablation (Lin et al., 2019). Another study identified important time windows in one modality (Porumb et al., 2020) with Grad-CAM (Selvaraju et al., 2020).

### 1.4. Existing multimodal explainability methods

As previously described, multiple explainability methods have been used with multimodal classifiers: FFS (Lajnef et al., 2015; Mellem et al., 2020), impurity (Lin et al., 2019), and ablation (Lin et al., 2019; Pathak et al., 2021). FFS is applicable to most classifiers. However, it requires retraining models many times, which is impractical for computationally intensive deep learning frameworks. Impurity is only applicable to tree-based classifiers. Lastly, ablation is, like FFS, also applicable to nearly any classifier and is easy to implement. In contrast to FFS, ablation is not computationally intensive. As such, of existing approaches, it is most useful for finding modality importance in deep learning classifiers.

### 1.5. Limitations of existing ablation approaches and novel alternatives

Ablation is related to perturbation-based methods like RISE (Petsiuk et al., 2018) or LIME (Ribeiro et al., 2016) that are frequently used in explainability for image classification and to methods like those presented in Thoret et al. (2021) that have been used in neuroscience applications. Importantly, ablation has a key weakness like all perturbation-based explainability methods. Specifically, perturbation methods can create out-of-distribution samples that lead to a poor estimates of modality importance (Molnar, 2018). Ablation involves (1) The substitution of a modality with neutral values (i.e., that do not give evidence for any one class) and (2) an examination of how that ablation affects the classifier. As such, when translating ablation to a new domain, it is important to consider how to set a modality to a neutral state while minimizing the likelihood out-of-distribution samples and features creation. Existing studies using ablation have replaced each modality with zeros (Lin et al., 2019; Pathak et al., 2021). However, zeroing out modalities creates samples that are highly irregular within the electrophysiology domain. In contrast, electrodes commonly return some line-related noise or, in instances when an electrode is not working properly, only return line-related noise. Line-related noise is found in electrophysiology data at 50 or 60 Hz due to the presence of lights, power lines and other electronics near recording devices. Because it is so often found in electrophysiology data, a classifier should learn to ignore it, and it should be neutral to the classifier. As such, line-related noise could offer a more reliable, electrophysiology-specific alternative to the zero-out ablation methods that have previously been applied.

While line noise-based ablation would be less likely to produce out-of-distribution samples and features than a zero-out ablation approach, it would still be at risk of doing so. Gradient-based feature attribution (GBFA) methods (Ancona et al., 2018) like Grad-CAM (Selvaraju et al., 2020), saliency (Simonyan et al., 2013), and LRP (Bach et al., 2015), in particular, offer an alternative to ablation that does not risk producing out-of-distribution samples. Additionally, local ablation methods, similar to saliency (Simonyan et al., 2013), show what features or time points make a sample more or less like the patterns learned by the classifier for a particular class. LRP shows what features or time points are actually used by the classifier for its classification and indicates their importance (Montavon et al., 2018).

### 1.6. Limitations of global explanations and proposal of novel local explainability approach

**FIGURE 1:**
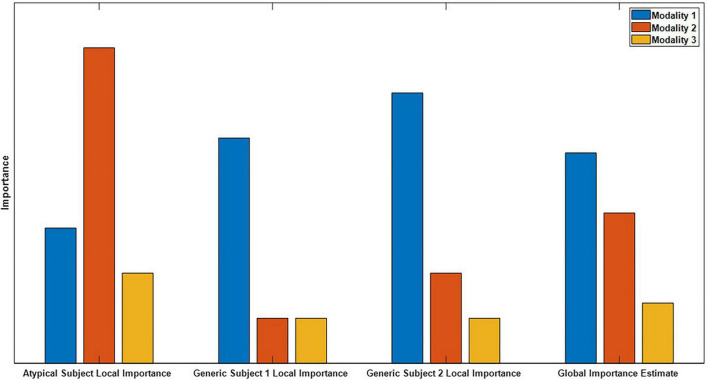
Example of global versus local importance with dummy data. From left to right, are 4 importance metrics: the importance for an atypical sample, the importance for two generic samples, and the global importance estimate that is formed by averaging the importance values for the individual samples. Note that modality two is very important for the atypical subject but not for the generic subject, so the presence of the atypical sample is hidden in the global importance. When thousands of samples from dozens of subjects are being analyzed, the presence of subgroups is easily obscured.

Global explainability methods identify the general importance of each modality to the classifier. In contrast, local methods provide higher resolution insight and indicate the importance of each modality to the classification of individual samples (Molnar, 2018). Global methods have inherent limitations relative to local methods, and existing multimodal explainability approaches have mainly been global. Importantly, as shown in Figure 1, global explanations obscure feature importance for individual samples and can obscure the presence of subgroups. Local explanations for many samples can be combined for higher level or global importance estimates (Ellis et al., 2021e,f). Because of this, they can also be analyzed on a subject-specific level that paves the way for the identification of personalized biomarkers. Furthermore, local explanations can be used to examine the degree to which demographic and clinical variables affect the patterns learned by a classifier for specific classes and features (Ellis et al., 2021c), which is a capacity that has not previously been exploited in multimodal classification. Local methods have been applied in a couple multimodal classification studies. In one study, authors ablated time points of an input sample and examined the effect on the classification of the sample (Pathak et al., 2021). In another study, authors used Grad-CAM to examine segments of a single modality (Porumb et al., 2020). Neither study identified the importance of each modality.

In the present study, we train a CNN for automated sleep stage classification using a publicly available dataset. We introduce a global ablation approach that is uniquely adapted for the electrophysiology domain (Ellis et al., 2021b). We then present a local ablation approach (Ellis et al., 2021c) and show how GBFA methods can be used for local insight into multimodal classifiers (Ellis et al., 2021a). With our local methods, we identify subject-level differences in modality importance that support the viability of the methods for personalized biomarker identification. We then use the local explanations in a novel analysis that provides insight into the patterns learned by the classifier related to the age, sex, and state of medication of subjects in our dataset (Ellis et al., 2021c,d).

## 2. Materials and methods

In this section, we describe our data, preprocessing, model architecture and training approach, and explainability methods.

### 2.1. Description of data

**FIGURE 2:**
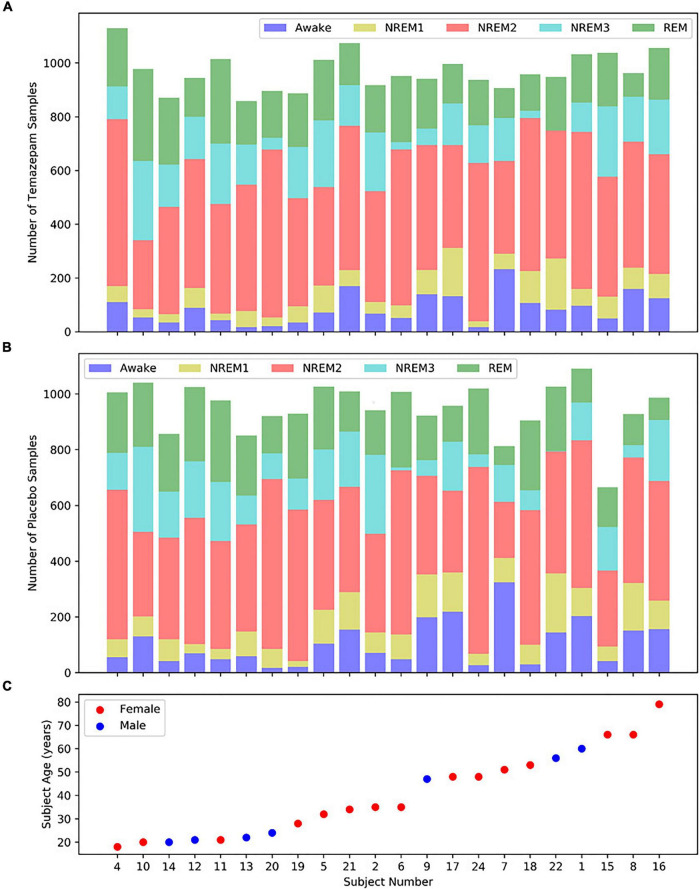
Distribution of samples and subject demographics. Panels **(A,B)** show the distributions of temazepam and placebo samples, respectively, for each subject. Panel **(C)** shows the age and sex of each subject, with the subjects arranged from youngest to oldest. Each panel shares the same *x*-axis.

We utilized Sleep Telemetry data from the Sleep-EDF Expanded Database (Kemp et al., 2000) on Physionet (Goldberger et al., 2000). The database has been used in previous studies (Vilamala et al., 2017; Rahman et al., 2018; Mousavi et al., 2019; Phan et al., 2019). Because the dataset was publicly available, no Internal Review Board approval was needed. The dataset has 44 approximately 9-h recordings from 22 subjects (15 female and 7 male) with primary sleep onset insomnia (Tuk et al., 1997). Subject age had a mean of 40.18 years and a standard deviation of 18.09 years. Figure 2 shows subject demographics. All subjects had two recordings–one following placebo administration and one following temazepam administration. Temazepam belongs to a class of drugs called benzodiazepines which amplify the effects of the neurotransmitter y-aminobutyric acid (GABA). GABA is inhibitory in nature and produces a calming effect on the brain (Griffin et al., 2013). It is often used to treat insomnia and affects electrophysiology activity. Each recording had data from 4 electrodes: 2 EEG, 1 EOG, and 1 EMG. Data was recorded at a 100 Hertz (Hz) sampling frequency. The EEG electrodes were FPz-Cz and Pz-Oz (Van Sweden et al., 1990), but like previous studies (Tsinalis et al., 2016b; Vilamala et al., 2017; Michielli et al., 2019; Mousavi et al., 2019; Phan et al., 2019), we used only Fpz-Cz. A 1-Hz marker indicated the presence of recording errors. Using the Rechtschaffen and Kales standard (Rechtschaffen and Kales, 1968), experts assigned 30-s epochs to seven categories: Movement, Awake, REM, NREM1, NREM2, NREM3, and NREM4. We merged NREM3 and NREM4 into a single NREM3 class (Iber et al., 2007), and we removed all samples containing movement or recording errors.

### 2.2. Description of data preprocessing

Based on the data annotation, we segmented the data into 30-s samples. Within each recording, we separately z-scored each electrode to improve cross-subject pattern identification. Our final dataset had 42,218 samples. The dataset was highly imbalanced with Awake, NREM1, NREM2, NREM3, and REM classes having 9.97, 8.53, 46.8, 14.92, and 19.78% of the dataset, respectively. We did not perform any filtering or reject data due to quality or noise issues.

### 2.3. Description of 1D-CNN

#### 2.3.1. Model architecture and training

**FIGURE 3:**
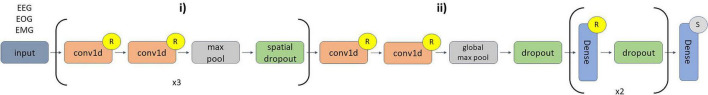
CNN architecture. Layers (i) of the diagram repeat 3 times. In (i) there are 6 1D-convolutional (conv1d) layers in total. The first two conv1d layers (number of filters = 16, kernel size = 5) are followed by max pooling (pool size = 2) and spatial dropout (rate = 0.01). The second two conv1d layers (number of filters = 32, kernel size = 3) are followed by max pooling (pool size = 2) and a spatial dropout (rate = 0.01). The third pair of conv1d layers (number of filters = 32, kernel size = 3) are followed by max pooling (pool size = 2) and spatial dropout (rate = 0.01). In (ii), the last two conv1d layers (number of filters = 256, kernel size = 3) are followed by global max pooling, dropout (rate = 0.01), and a flatten layer. The first two dense layers (number of nodes = 64) have dropout rates of 0.1 and 0.05, respectively. The last dense layer has 5 nodes. The inputs sample shape was 3,000 time points × 3 modalities, and no layers used zero padding during convolution. An “R” or an “S” indicates that a layer is followed by ReLU or Softmax activation functions, respectively.

We adapted a CNN architecture initially developed for EEG classification (Youness, 2020). The architecture is shown in Figure 3. We implemented the architecture in Keras (Chollet, 2015) with a TensorFlow (Abadi et al., 2016) backend. We used 10-fold cross-validation with a random 17-2-3 subject training-validation-test split each fold. We used class-weighted categorical cross entropy loss to account for class imbalances. We used a batch size of 100 with shuffling after each epoch. We used the Adam optimizer (Kingma and Ba, 2015) with an adaptive learning rate. Starting at a learning rate of 0.001, the step size decreased by a factor of 10 if validation accuracy did not improve within a 5-epoch window. We used early stopping to end training if validation accuracy plateaued for 20 epochs with a maximum of 100 epochs and used model checkpoints to select the model from each fold that obtained the best validation accuracy. We used the selected models for testing and explainability.

#### 2.3.2. Model performance evaluation

When evaluating model test performance, we sought to account for class imbalances. We calculated the precision, recall, and F1 score for each class. We calculated the mean and standard deviation of the metrics across folds.


$$P\,r\,e\,c\,i\,s\,i\,o\,n=\frac{T\,r\,u\,e\,P\,o\,s\,i\,t\,i\,v\,e}{T\,r\,u\,e\,P\,o\,s\,i\,t\,i\,v\,e+F\,a\,l\,s\,e\,P\,o\,s\,i\,t\,i\,v\,e}$$

$$R\,e\,c\,a\,l\,l=\frac{T\,r\,u\,e\,P\,o\,s\,i\,t\,i\,v\,e}{T\,r\,u\,e\,P\,o\,s\,i\,t\,i\,v\,e+F\,a\,l\,s\,e\,N\,e\,g\,a\,t\,i\,v\,e}$$

F⁢1=2*P⁢r⁢e⁢c⁢i⁢s⁢i⁢o⁢n⁢R*⁢e⁢c⁢a⁢l⁢lP⁢r⁢e⁢c⁢i⁢s⁢i⁢o⁢n+R⁢e⁢c⁢a⁢l⁢l

### 2.4. Description of global ablation approaches

We applied two global ablation approaches to estimate class-specific modality importance. We presented a novel global ablation approach that is uniquely adapted to the electrophysiology domain (Ellis et al., 2021b) and compared our approach to a standard approach that has been used in previous studies (Lin et al., 2019; Pathak et al., 2021).

Generally, ablation takes place after model training. It involves replacing a feature or modality with zeros during model evaluation and examining the change in model performance following the loss of the information in that modality or feature. The importance of the replaced feature or modality to the model is directly related to the decrease in model performance associated with its loss. A feature *f1* is more important to a model than a feature *f2* if the effect of ablating *f1* is greater than the effect of ablating *f2.* Our standard ablation approach had several key steps (1). We calculated a confusion matrix (i.e., a model performance estimate) for the test data in a fold (2). We replaced a modality *m* with zeros across all test samples in the fold (i.e., ablation) (3). We calculated a confusion matrix for the classifier on the test data with the replaced modality *m* (4). We calculated the percent change (PCG) in samples assigned to each classification group following ablation (i.e., effect of ablation). Example classification groups include NREM1 samples classified as REM, NREM2 samples classified as NREM3, and REM samples classified as REM (5). We repeated steps 2 through 4 for each modality *m* (6). We repeated steps 1 through 5 for each fold.


P⁢C⁢G=

$$100*\frac{N\,u\,m\,b\,e\,r\,o\,f\,M\,o\,d\,i\,f\,i\,e\,d\,S\,a\,m\,p\,l\,e\,s-N\,u\,m\,b\,e\,r\,o\,f\,U\,n\,m\,o\,d\,i\,f\,i\,e\,d\,S\,a\,m\,p\,l\,e\,s}{N\,u\,m\,b\,e\,r\,o\,f\,U\,n\,m\,o\,d\,i\,f\,i\,e\,d\,S\,a\,m\,p\,l\,e\,s}$$

We propose an ablation approach for multimodal electrophysiology analysis that involves replacing modalities in a way that mimics line-related noise. This approach involves all of the steps detailed previously. However, we modify Step 2 of the ablation process. Instead of replacing modality *m* with zeros, we replace modality *m* with a combination of a sinusoid and Gaussian noise. We use a sinusoid with a frequency of 50 Hz and an amplitude of 0.1, and the Gaussian noise had a mean of 0 and standard deviation of 0.1. To determine whether our line-related noise approach yielded results significantly different from standard ablation, we performed a series of two-tailed t-tests. Within each modality, we compared the importance values in each classification group for each method across folds.

### 2.5. Description of novel local ablation approach

We developed a local ablation approach for insight into modality importance (Ellis et al., 2021c). Our novel ablation approach is similar to the global approach described in the previous section (1). We obtained the top-class probability for a sample (2). We ablated a modality in that sample (3). We obtained the classification probability of the modified sample for the original top class (4). We computed the percent change in classification probability (5). We repeated steps 2 through 4 for each modality (6). We repeated steps 2 through 5 for each sample (7). We repeated steps 2 through 6 for each fold.


P⁢C⁢G=

$$100*\frac{M\,o\,d\,i\,f\,i\,e\,d\,S\,a\,m\,p\,l\,e\,P\,r\,o\,b\,a\,b\,i\,l\,i\,t\,y-U\,n\,m\,o\,d\,i\,f\,i\,e\,d\,S\,a\,m\,p\,l\,e\,P\,r\,o\,b\,a\,b\,i\,l\,i\,t\,y}{U\,n\,m\,o\,d\,i\,f\,i\,e\,d\,S\,a\,m\,p\,l\,e\,P\,r\,o\,b\,a\,b\,i\,l\,i\,t\,y}$$

Because there were no preexisting local approaches, we compared our local ablation results to the global ablation results. In addition to generating local visualizations of our results, we estimated global importance by calculating the mean absolute percent change in classification probability for each fold.

### 2.6. Description of layer-wise relevance propagation analysis

Layer-wise relevance propagation (LRP) (Bach et al., 2015) was first developed for image analysis but has since been used in electrophysiology (Sturm et al., 2016) and other neuroscience domains (Yan et al., 2017; Thomas et al., 2018; Ellis et al., 2021e). We implemented LRP with the Innvestigate library (Alber et al., 2019). LRP is a local explainability method but has can be used for global importance estimates (Ellis et al., 2021a,e). LRP involves several steps (1). A sample is passed through a network and assigned a class (2). A total relevance of 1 is placed at the output node of the assigned class (3). The relevance is propagated through the network to the input sample space with relevance rules. Importantly, the total relevance is conserved when propagated through the network such that the total relevance assigned to the sample space should equal the original total relevance. LRP can output both negative and positive relevance. Negative relevance indicates features that support a sample being classified as a class other than that which it was assigned. Positive relevance indicates features that support a sample being classified as its assigned class. In our study, we used the ε-rule and αβ-rule. The equation below shows the ε-rule.


$$R_{j}=\sum_{k}\frac{a_{j}\,w_{j\,k}}{\varepsilon +\sum_{0,j}a_{j}\,w_{j\,k}}\,R_{k}$$

where *k* indicates a node that is one of *k*nodes in a layer deeper in a network and *j* indicates a node in the layer to which relevance is being propagated. *R_k_* indicates the total relevance assigned to a node in a deeper layer, and *R_j_* indicates the total relevance that will be assigned to a node in a shallower layer. The variables *a_j_* and *w*_*jk*_ indicate the activation output of the layer j and the value of the weight connecting the node in layer *j* and node in layer *k*. The numerator indicates a portion of the effect that the node in layer *j* has upon the node in layer *k*, and the denominator indicates the total effect of all nodes in layer *j* upon the node in layer *k*. This combined with the summation Σ_*k*_ indicates that the relevance assigned to the node in layer *j* is the sum of the fraction of the effect of the node in layer *j* upon all of the nodes in layer *k* multiplied by their respective relevance. The term “ε” enables relevance to be filtered when propagated through the network. A larger ε shrinks the amount of relevance propagated backward for nodes that would otherwise be assigned low relevance. In effect, this reduces the noisiness of the explanations. We used the ε-rule with an ε of 0.01 and 100.

The αβ-rule is shown in the equation below,


$$R_{j}=\sum_{k}\left(\alpha \,\frac{{\left(a_{j}\,w_{j\,k}\right)}^{+}}{\sum_{0,j}{\left(a_{j}\,w_{j\,k}\right)}^{+}}-\beta \,\frac{{\left(a_{j}\,w_{j\,k}\right)}^{-}}{\sum_{0,j}{\left(a_{j}\,w_{j\,k}\right)}^{-}}\right)\,R_{k}$$

where the relevance is split into positive and negative portions. The variables α and β control how much positive and negative relevance are propagated backward, respectively. In our study, we only propagated positive relevance (i.e., α = 1, β = 0).

In our analysis, we generated a global estimation of importance by calculating the percent of absolute relevance assigned to each modality. We computed this value for each classification group in each fold. We also visualized how the percent of relevance varied over time.

### 2.7. Description of statistical analyses

We performed a series of statistical analyses with the local ablation and LRP (ε-rule with ε = 100) explanations for insight into the effects of demographic and clinical variables upon the classifier. To account for interaction effects, we trained an ordinary least squares regression model with age, medication, and sex as the independent variables and with the absolute importance (i.e., percent change in activation for local ablation and relevance for LRP) for a modality and classification group as the dependent variable. For LRP, we used the percent of absolute relevance assigned to each modality for each sample. After training the model, we obtained the resulting coefficients and *p*-values for each class. The sign of the coefficients identified the direction of the importance difference. After obtaining *p*-values, we performed false discovery rate (FDR) correction (α = 0.05) with the 25 *p*-values (i.e., 5 classes × 5 classes) associated with each clinical or demographic variable to account for multiple comparisons.

## 3. Results

Here, we describe our model performance, explainability, and statistical analysis results.

### 3.1. Model performance results

Table 1 shows the mean and standard deviation of the precision, recall, and F1 score for each class. The model had highest F1 scores for NREM2 and Awake. Possibly because of its smaller sample size, NREM1 had the lowest classification performance across all metrics. While performance for NREM3 and REM was not as high as for NREM2 and Awake for most metrics, the classifier still performed well for both classes.

**TABLE 1:** Classification performance results.

	Awake	NREM1	NREM2	NREM3	REM
F1	71.25 ± 05.15	39.86 ± 07.19	73.28 ± 04.76	64.15 ± 15.25	65.92 ± 06.28
Precision	72.25 ± 07.12	36.20 ± 03.98	79.35 ± 03.92	56.78 ± 18.35	69.04 ± 07.14
Recall	70.90 ± 07.02	46.28 ± 13.52	68.71 ± 08.51	78.22 ± 10.24	63.26 ± 06.69

### 3.2. Global explainability results

**FIGURE 4:**
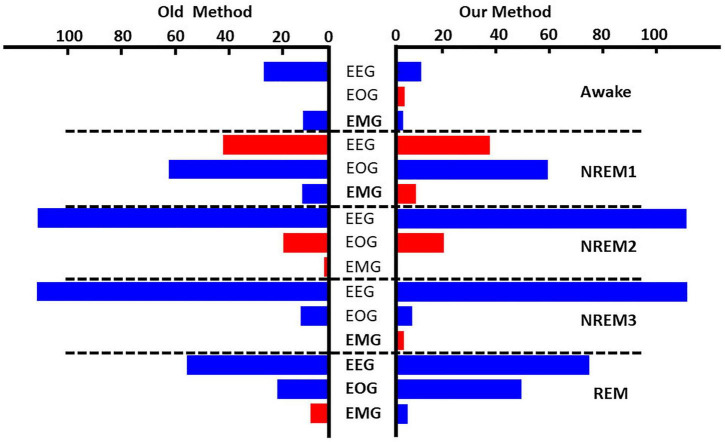
Global ablation results for correctly classified samples. The leftmost and rightmost plots show the results for zero-out and noise-related ablation, respectively. Horizontal dashed lines separate importance for each sleep stage. The *x*-axis indicates the mean percent change in samples across folds. Blue and red bars indicate a negative and positive percent change, respectively. Bolded modalities have significant differences (*p* < 0.05) between results for the two methods. Across methods, EEG was most important for all classes except NREM1.

**FIGURE 5:**
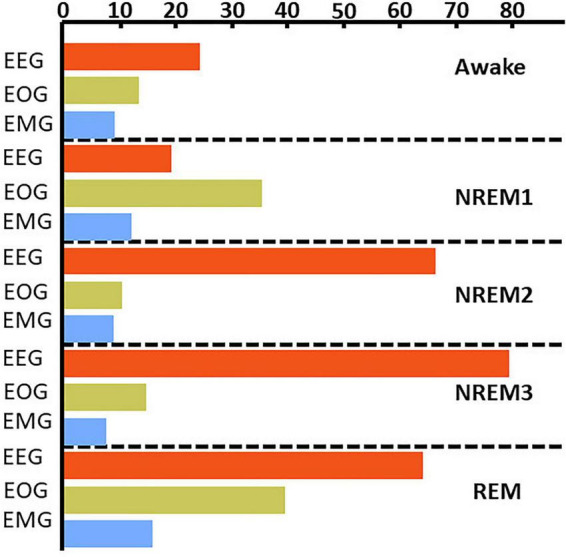
Local ablation results showing estimation of global importance for correct classification groups. Within each fold, we calculated the mean absolute percent change in activation for the perturbation of samples in each classification group. We then calculated the median value across folds. The *x*-axis indicates the values associated with that percent change in activation. The bar plots show the results for the correct classification group. EEG, EOG, and EMG importance values are shown in red, green, and blue, respectively. EEG was most important for all classes except NREM 1.

**FIGURE 6:**
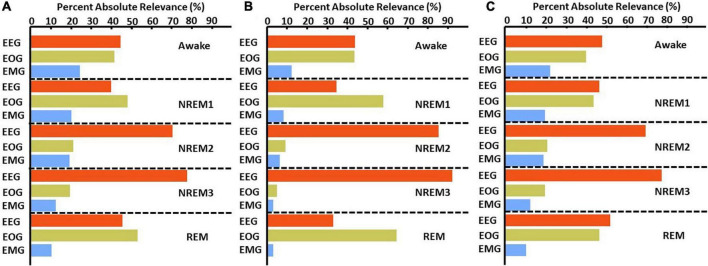
LRP global modality importance results for correct classification groups. We calculated the percent of absolute relevance for each modality across samples within each classification group. We then calculated the median value across folds. Panels **(A–C)** show results for the αβ-, ε- (ε = 100), and ε-rules (ε = 0.01), respectively. Red, green, and blue bars are for EEG, EOG, and EMG, respectively. EEG was generally most important.

Figure 4 and Supplementary Figure 1 show the results comparing noise-related global ablation with the typical zero-out global ablation approach for correct classification groups and all classification groups, respectively. Interestingly, the methods generally agreed upon the relative importance of each modality. However, there were multiple significant differences in which our method seemed to amplify the effect of perturbation more than the typical zero-out approach. Figure 5 and Supplementary Figure 2 show our local ablation results estimating global modality importance for correct classification groups and all classification groups, respectively. Figure 6 and Supplementary Figure 3 show the LRP results for correct classification groups and all groups, respectively. We compared the relative magnitude of the estimates across methods. Across methods, EEG was generally most important. For Awake/Awake, all three methods found that EEG was most important, though LRP magnified the importance of EOG and EMG relative to EEG more than local ablation. For NREM1/NREM1, two LRP rules and local and global ablation found that EOG was most important, followed by EEG. NREM2/NREM2 and NREM3/NREM3 results were similar across methods. EEG was most important, followed by EOG and EMG. For REM/REM, only LRP ε-rule (ε = 0.1) agreed with local and global ablation regarding the relative modality importance. They identified the order of importance as EEG, EOG, and EMG. Many incorrect classification groups had similar distributions of relative importance across methods. However, some groups had different importance distributions. NREM1/Awake generally had greater EOG than EEG relevance for LRP but not for ablation. NREM2/NREM1 had less EOG than EEG relevance for LRP but not for ablation. Awake/REM and NREM1/REM had more EEG than EOG importance for local ablation but not for LRP. Global ablation found that EEG and EOG importance for Awake/REM varied according to the global ablation approach. Additionally, global ablation found that EEG had greater importance than EOG for NREM1/REM.

### 3.3. Subject-level local explainability results over time

**FIGURE 7:**
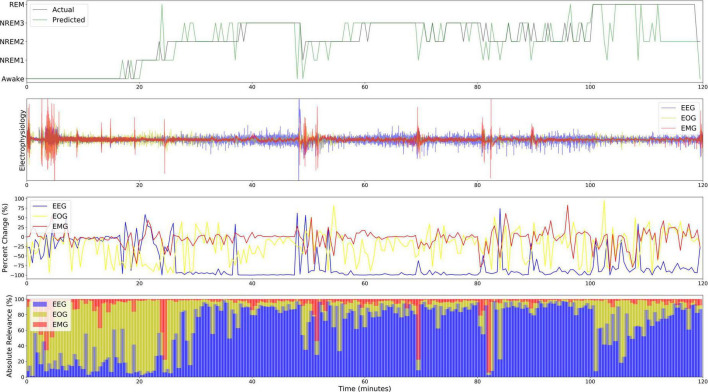
Local explanations over a 2-H sleep cycle from subject 12. The first, second, third, and fourth panels show the actual and predicted classes, electrophysiology activity, local ablation results, LRP results (ε = 100). Unlike the global results, EOG is more important for Awake than EEG from 0 to 20 min.

Figure 7 shows both the local ablation and LRP results over the first 2 h of a recording from Subject 12. Both methods showed similar trends in modality importance over time. They both showed lower levels of EEG and higher levels of EOG importance during Awake and NREM1 periods and showed a transition to higher EEG and lower EOG importance for NREM2, NREM3, and REM. However, LRP often seemed to more closely correspond with changes in electrophysiology activity. For example, between 60 and 80 min, EMG activity spiked, and a misclassification resulted. In this case, LRP more clearly indicated that the change affected the classification. Additionally, for NREM periods from 30 to 100 min, EEG relevance had greater variation relative to the that of other modalities than the local ablation results.

### 3.4. Statistical analysis of effects of clinical and demographic variables upon local explanations

**FIGURE 8:**
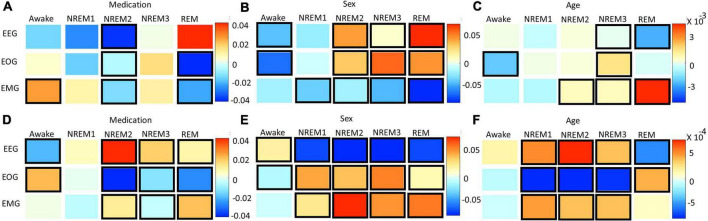
Effects of clinical and demographic variables for correct classification groups. Panels **(A–C)** show effects of medication, sex, and age, respectively, on the local ablation results. Panels **(D–F)** show effects of medication, sex, and age, respectively, on the LRP results. The *x*- and *y*-axes indicate the predicted class and modality, respectively. The heatmaps show the regression coefficient values. Bolded boxes show significant effects (*p* < 0.05). A positive medication coefficient indicates that temazepam samples had more importance than placebo samples. A positive subject sex coefficient indicates that female samples had more importance than male samples, and a positive age coefficient indicates that importance increased with age. Note that sex had more significant relationships than the other variables.

Figures 8A–C and Supplementary Figure 4 show the results for the statistical analysis examining the effects of medication, sex, and age upon the local ablation explanations for correct classification groups and all groups, respectively. Figures 8D–F and Supplementary Figure 5 show the results for the analysis applied to the LRP relevance. Many effects were consistent between the two methods. Across both methods, subject sex had relationships with more correct classification group modality pairs than either medication or age. The importance of EEG for Awake/Awake was less in temazepam than placebo samples and was more in temazepam than placebo samples for REM/REM. Samples assigned to NREM3 also generally had more EEG importance for temazepam than placebo samples. For EOG, most groups with significant relationships with medication had more importance across both methods in placebo than in temazepam samples. In REM/REM, EOG importance was higher in placebo than temazepam samples. NREM2/NREM1 and NREM3/NREM2 had more EMG importance in temazepam than placebo samples for both methods. REM/NREM2 had less EMG importance in temazepam than placebo samples for both methods.

For the effects of sex on EEG importance, the two methods provided similar results in a couple cases: (1) NREM1/NREM2 and (2) NREM2/NREM1. However, different effects occurred in many cases: (1) NREM2/NREM2, NREM2/NREM3, and NREM2/REM, (2) NREM3/NREM3 and NREM3/NREM2, and (3) REM/REM and REM/NREM2. For EOG, correctly classified Awake, NREM2, NREM3, and REM had similar differences in importance between males and females, and the changes in importance for many classification groups were similar across methods. For EMG and sex, the differences in importance between male and female were similar in a few cases (e.g., Awake/REM, NREM1/NREM3, and REM/NREM3).

Across methods, age affected the importance assigned to modalities. For EEG, there were similar effects of age: (1) Awake/NREM3, (2) NREM2/NREM1 and NREM2/REM, and (3) REM/REM and REM/Awake. Many groups with different results across methods were insignificant for local ablation. For EOG, there were differences between many classification groups. However, NREM1/REM and NREM2/REM, and NREM3/NREM1 had similar results across methods. For EMG, there were many similarities in the effect of age upon the explanations of the methods: (1) NREM1/NREM2, (2) NREM2/NREM2 and NREM2/Awake, (3) NREM3/NREM3, and (4) REM/NREM3.

## 4. Discussion

In this section, we discuss the broader implications of our methods. We then discuss our results within the context of sleep literature and discuss future research directions.

### 4.1. Implications of novel explainability methods beyond sleep stage classification

In this study, we present a series of novel multimodal explainability methods. Our global ablation method is uniquely adapted to multimodal electrophysiology data. Additionally, its finding of enhanced effects relative to a zero-out approach highlights the utility of using domain-specific perturbations. Our local ablation approach is the first local multimodal explainability method that provides insight into the importance of each modality. By examining the change in output activation following ablation, it also shows how ablation or perturbation could be used to obtain local explanations across a variety of explainability problems beyond multimodal explainability. It is, in its current state, only applicable to electrophysiology data, but it could be easily adapted to other domains. We also show, for the first time, how gradient-based methods can be used to find modality importance both locally and globally. Because they do not perturb data, GBFA methods could offer a more reliable approach than ablation. Importantly, our ablation and gradient-based methods could each be better suited to different models. Unlike gradient methods, ablation is easily applicable to all deep learning classification frameworks. For example, our ablation methods would be more effective for long short-term memory (LSTM) networks than our LRP approach. While LRP can be applied to long short-term memory networks (Arras et al., 2017), doing so can be challenging, especially given that LSTMs are not supported in the Innvestigate library, and problems can arise with model gradients. As such, it is generally easier to implement for most CNN or multilayer perceptron architectures (Ellis et al., 2021e). It is also important to note that the insights provided by ablation and LRP are slightly different. Ablation gives a quantitative estimate of the sensitivity of the model to the loss of information in a modality. In contrast, LRP gives an estimate of the reliance of the model upon a given modality in the classification of a specific sample. Our local methods could help identify subject-specific electrophysiology biomarkers for personalized medicine. Additionally, our analysis of the relationship between local explanations and demographic and clinical variables offers a way to gain insight into the effects of variables that are not explicitly included in the training data and has implications beyond multimodal explainability. Model developers could use it to better understand how aspects of data are affecting their models. Additionally, it could increase physicians’ and other relevant decision-makers’ trust of deep learning-based systems and jointly increase the likelihood of clinical adoption. It could also help scientists develop hypotheses for novel biomarkers.

### 4.2. Classification performance

Our classifier performed well but slightly below state-of-the-art classifiers (Chambon et al., 2017). Performance was worst on NREM1. This makes sense given that NREM1 is the smallest class and can be similar to Awake and REM (Iber et al., 2007; Tsinalis et al., 2016a). NREM1 classification has often been relatively poor in previous studies (Tsinalis et al., 2016a; Supratak et al., 2017; Chambon et al., 2018; Michielli et al., 2019). Although Awake and NREM1 had similar numbers of samples, the classifier performed much better on Awake. Given that Awake EEG and EOG have features that are very different from those of NREM and that Awake EMG is different from REM EMG (Iber et al., 2007), it makes sense that it would be easier to classify Awake samples. Similar to previous studies (Chambon et al., 2017; Supratak et al., 2017), the precision and F1 score, but not recall, were highest for NREM2.

### 4.3. Global results

Across methods, EEG was most important for identifying Awake, NREM2, NREM3, and REM. In contrast, EOG played a greater role in the correct classification of NREM1 samples. EMG was not very important to the classification of any stage. This result is not atypical, as previous studies have shown that using EEG and EMG does not greatly improve classification performance for Awake, NREM2, and NREM3 relative to only using EEG (Kim and Choi, 2018). While global explanations for correct classification groups were similar across methods, explanations for incorrect groups tended to differ across methods.

### 4.4. Subject-level local ablation and LRP results over time

The two local approaches had similar results for the 2-h period of explanations that we output. In contrast to the global explanations, EOG was particularly important during Awake periods. This suggests that subject or subgroup-specific patterns of EOG activity exist within the Awake class that are obscured by global methods. It also supports existing findings that EEG alone did not discriminate between Awake, NREM1, and REM as effectively as EEG with EOG and EMG (Estrada et al., 2006). Additionally, previous studies have found that EOG is particularly important for identifying Awake (Pettersson et al., 2019) and can yield comparable classification performance to EEG (Ganesan and Jain, 2020). In contrast, EEG was important for discriminating NREM and REM samples, which makes sense given that NREM and REM EEG differ greatly (Iber et al., 2007). It is interesting that the subject had higher Awake EOG than EEG importance. Globally, EEG tended to be more important for Awake than EOG. Moreover, visualizing the results over time enabled us to obtain higher resolution insight into the classifier than global visualization. For example, EMG importance for the subject spiked for incorrectly classified samples, which suggests that EMG adversely affected model performance for some subjects.

### 4.5. Statistical analysis of effects of clinical and demographic variables upon local explanations

Interestingly, sex has relationships with more modality correct classification group pairs than either medication or age, which could indicate that subject sex had stronger effects on the patterns learned by the classifier than the other variables. This is potentially attributable to the imbalance of male and female subjects. Subject sex seemed to affect the NREM2 EEG patterns learned by the classifier. This reflects established sleep science. Namely, adult women can have greater slow-wave EEG activity in NREM sleep stages than men (Mourtazaev et al., 1995; Ehlers and Kupfer, 1997), and, in general, there are differences in the EEG activity of men and women (Armitage and Hoffmann, 2001; Bučková et al., 2020). While sex was associated with the correct classification of NREM1, sex may have adversely affected the EEG patterns learned by the classifier for Awake, NREM1, NREM2, and REM. Whereas the effects of sex on EEG was more associated with incorrect classification, both explainability methods indicated that sex likely affected the EOG patterns learned by the classifier for the correct classification of Awake, NREM2, NREM3, and REM. This highlights the possibility of EOG sex differences across most sleep stages. Our literature review has uncovered no studies on the effects of sex upon EOG in sleep, so our results could prompt future studies on this topic. Both methods indicated that sex affected the EMG patterns learned for incorrectly classified samples. Medication affected the EEG of Awake, NREM3, and REM similarly, with both methods. Previous studies have shown that benzodiazepines like temazepam (Bastien et al., 2003) and other medications (Chalon et al., 2005) can have significant effects on EEG sleep stages and that temazepam, in particular, can greatly affect REM (Pagel and Farnes, 2001). Other studies have shown similar effects in monkey EEG (Authier et al., 2014). Our results also showed that medication significantly affected the patterns learned for REM EOG. Interestingly, medication may have been related to the learning of EMG patterns that contributed to incorrect NREM classification. The inconsistent effects of medication upon EMG could fit with previous studies that purportedly analyzed EMG sleep data in monkeys but did not report any effects of medication (Authier et al., 2014). The effects of age on sleep are well characterized (Mourtazaev et al., 1995; Ehlers and Kupfer, 1997; Boselli et al., 1998; Chinoy Frey et al., 2014; Luca et al., 2015). In our study, age seemed to affect the EEG patterns learned for REM like in Landolt and Borbély (2001). However, age was also related to the learning of EEG patterns for multiple incorrect classification groups. This suggests that the model did not fully learn to address the underlying effects of age upon EEG across sleep stages. Interestingly, age had inconsistent effects upon the EOG patterns learned by the classifier. Age seemed to affect EMG patterns for NREM1 and NREM2.

### 4.6. Limitations and next steps

Future studies might compare differences in importance across more subjects, which could help identify personalized sleep stage biomarkers (Porumb et al., 2020). For our line-related noise global ablation approach, we used a combination of a 50-Hz sinusoid and Gaussian noise. This approach provided a useful proof-of-concept and is viable for use in future studies. However, it only provides a simple simulation of line noise. Line noise can, in practice, have a more complex power spectral density around 50 Hz. In this study, we used a simple CNN classifier, which made the implementation of LRP straightforward. However, using a simple CNN classifier also contributed to classification performance that was high but below the state of the art. Future studies with advanced classifiers might use the analyses that we employed to assist with the discovery of biomarkers and formulation of novel hypotheses related to sleep and other domains. Our classifier was originally developed for EEG sleep stage classification. As such, the architecture may not be optimized for EOG and EMG feature extraction. While this does not adversely affect the quality of our explainability results, it prevents generalizable claims regarding the importance of one modality over another. Additionally, other GBFA methods could potentially replace LRP for multimodal explainability. Metrics like those presented in Samek et al. (2017a), Petsiuk et al. (2018) could help rate the quality of each explainability method, and future studies might enhance LRP explanation quality by applying different relevance rules to different parts of a network (Samek et al., 2017b). Additionally, while our analysis of relationships between local explanations and clinical and demographic variables was insightful, future studies might perform a variety of other analyses on local explanations (Thoret et al., 2022). For example, they might cluster local explanations to identify subtypes of individuals or compute measures that quantify aspects of the temporal distribution of importance. Lastly, our dataset was only composed of data from 22 participants. As such, the generalizability of the conclusions that can be drawn from our analysis of the relationship between the local explanations and clinical and demographic variables is somewhat limited. Nevertheless, the analysis represents a novel approach for the domain and offers inspiration as a starting point for future studies in the field.

## 5. Conclusion

In this study, we use sleep stage classification as a testbed for developing multimodal explainability methods. After training a classifier for multimodal sleep stage classification, we present a series of novel multimodal explainability methods. Up to this point, relatively few studies in the domain of multimodal classification have involved explainability, which is particularly concerning for clinical settings. Our global ablation method is uniquely adapted to electrophysiology classification. Our local ablation approach is the first local multimodal ablation method, and our GBFA approach offers an alternative to ablation that has not previously been used for modality importance. We find that EEG was most important to the identification of most sleep stages while EOG was most important to the identification of NREM1. We show how local methods can help identify differences in subject-level explanations that could potentially be used to identify personalized biomarkers in future studies. Importantly, we also developed a novel analysis approach and found that subject sex had more significant relationships with patterns learned by the classifier relative to other clinical and demographic variables. More broadly, the approach could help illuminate the effects of those variables upon different classes (e.g., sleep stages or disease conditions). Our study enhances the level of insight that can be obtained from the typically black-box models of the growing field of multimodal classification and has implications for personalized medicine and the eventual development of multimodal clinical classifiers.

## Data availability statement

Publicly available datasets were analyzed in this study. This data can be found here: https://www.physionet.org/content/sleep-edfx/1.0.0/.

## Author contributions

CE helped with the conception of the manuscript, performed the analyses, wrote the manuscript, and edited the manuscript. MS helped with figure creation, writing, and editing the manuscript. RZ and DC helped perform analyses and edited the manuscript. MW and RM helped with conception of the manuscript and edited the manuscript. VC helped with the conception of the manuscript, edited the manuscript, and provided funding for the manuscript. All authors contributed to the article and approved the submitted version.
