# Supplementary material for: Novel methods for elucidating modality importance in multimodal electrophysiology classifiers
Source: Front Neuroinform. 2023 Mar 15;17:1123376. doi: 10.3389/fninf.2023.1123376 (PMC10050434; doi:10.3389/fninf.2023.1123376)
Supplement: Supplementary file 1 [file Data_Sheet_1.DOCX]

Supplementary Material

## Supplementary Information - Characteristic Features of Sleep Stages

Typical sleep stage classification approaches involve the classification of 5 stages: Awake, rapid eye movement (REM), non-REM1 (NREM1), NREM2, and NREM3. Clinicians use the American Academy of Sleep Medicine (AASM) manual (15) to identify the stages, and the stages are clearly described in other sleep staging studies (20)(21). According to the AASM manual (15), Awake periods are characterized by α band (8 – 13 Hz) electroencephalogram (EEG) activity in occipital brain regions when a participant’s eyes are closed. When a participant’s eyes are open, Awake periods are characterized by eye blinks, rapid eye movements, reading eye movements (i.e., slow movements followed by a burst of movement in the opposite direction), and strong levels of electromyogram (EMG) activity. NREM1 periods are characterized by slow eye movements, θ band (4 – 7 Hz) EEG activity, and vertex sharp waves (i.e., large V-shaped waves with a duration of less than 0.5 seconds). They have EMG activity at lower levels than Awake periods. NREM2 is principally characterized by EEG activity. It has K-complexes (i.e., sharp negative decreases in signal amplitude followed immediately by sharp increases in positive signal amplitude) and sleep spindles (i.e., trains of waves between 11 – 16 Hz). NREM2 typically has no electrooculogram (EOG) activity. It typically has less EMG than Awake periods and can have levels as low as REM. NREM3 mainly consists of δ (0.5 – 2 Hz) EEG activity. NREM3 typically has little or no EOG activity. It has less EMG activity than NREM2 and can have EMG activity as low as REM. REM is characterized by rapid eye movements in the EOG, periods of little or no EMG activity followed by brief irregular bursts of activity, and sharp triangular EEG activity. Each stage is characterized by distinct EEG, EOG, and EMG activity.

## Supplementary Figures


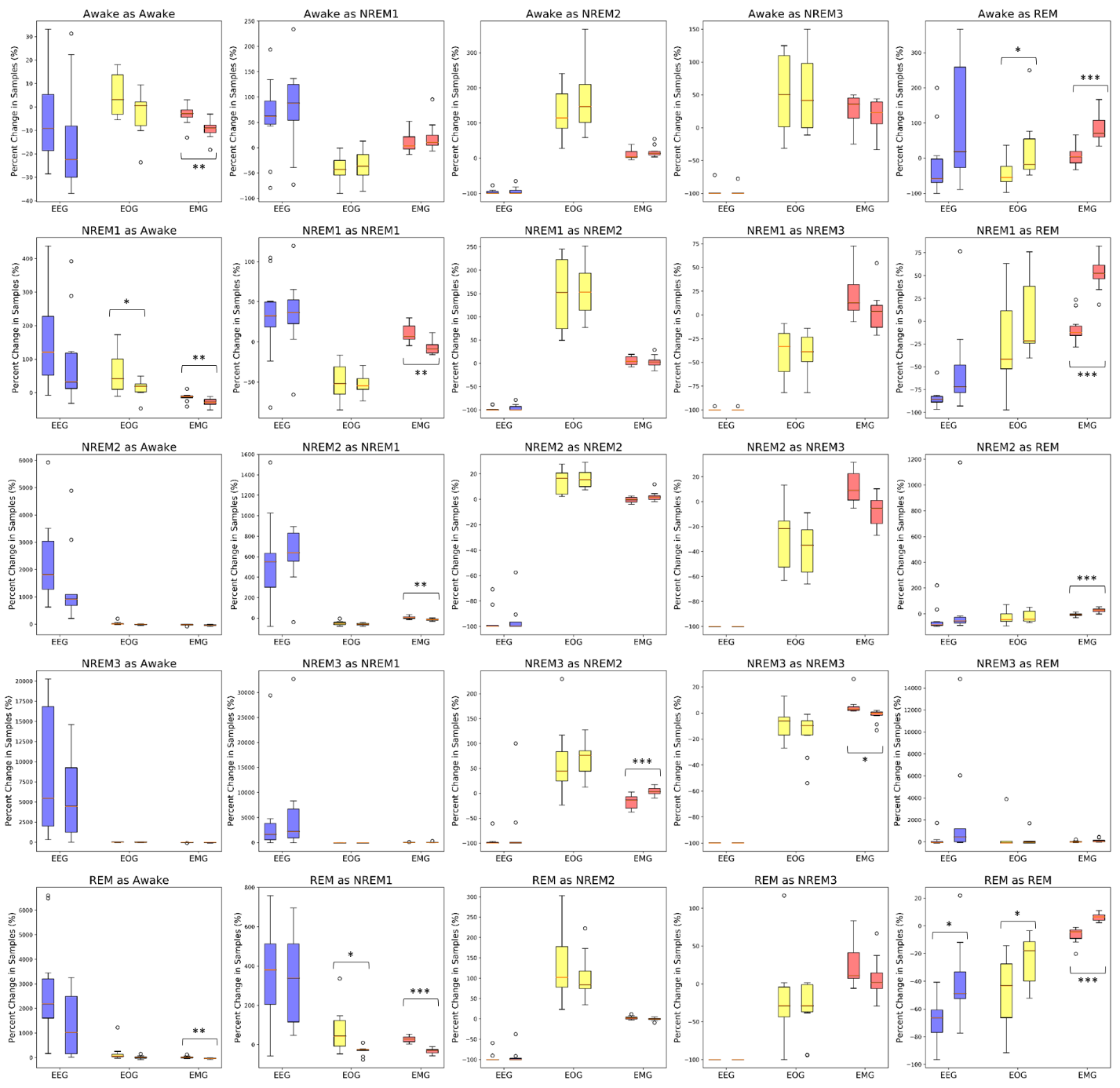


**Supplementary Figure 1.** Global Ablation Explainability Results for All Classification Groups. The above boxplots show the percent change in samples assigned to each classification group following perturbation for each fold. Blue, yellow, and red boxes indicate EEG, EOG, and EMG perturbation, respectively. The leftmost box of each pair shows the results for our noise-related ablation method. The rightmost box is for the typical zero-out ablation approach. Some pairs of boxes are labeled with *, **, or ***, which correspond to a significance value of p < 0.05, p < 0.01, and p < 0.001, respectively.


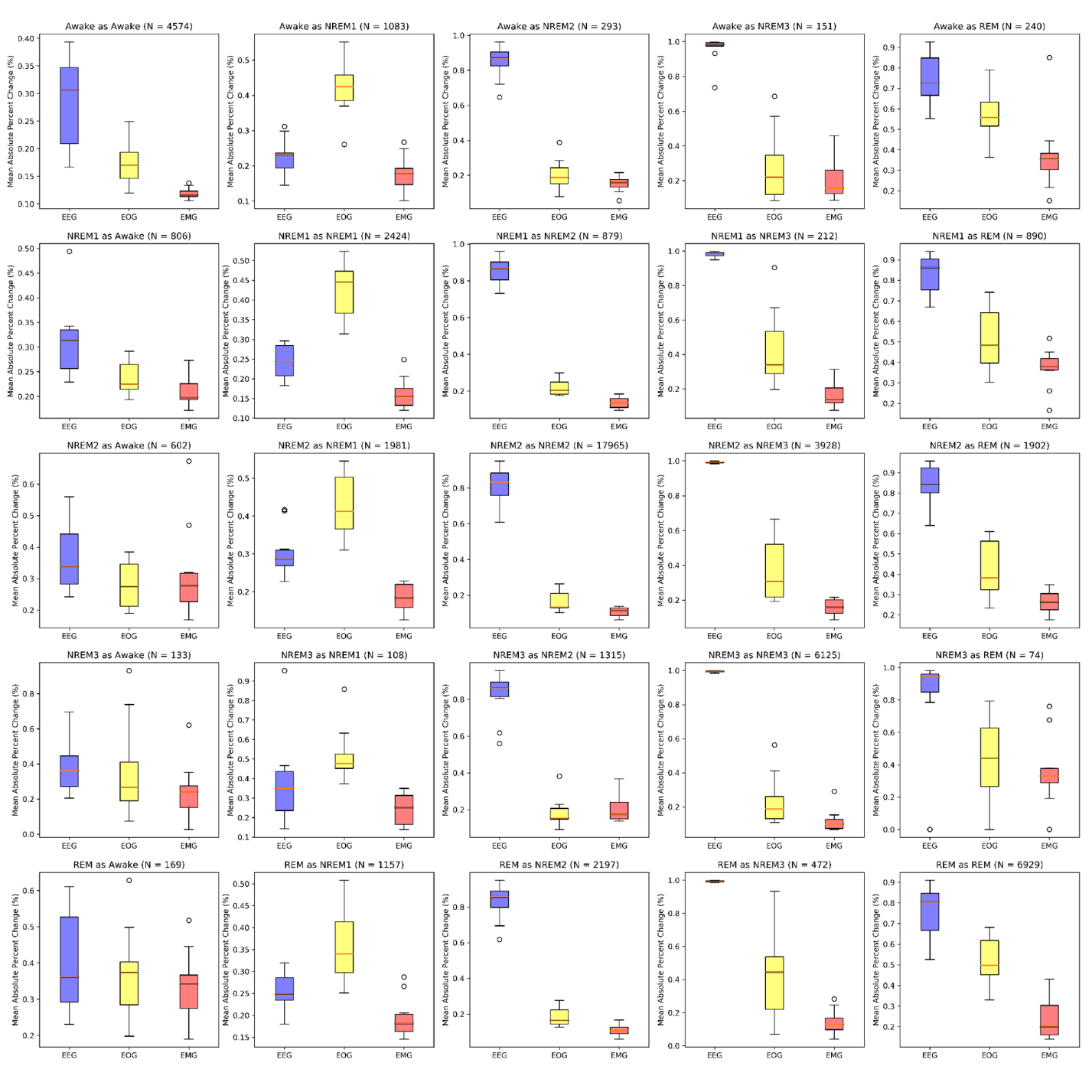


**Supplementary Figure 2.** Local Ablation Results Showing Global Estimation of Modality Importance for All Classification Groups. The above boxplots show the percent change in activation following perturbation for samples in each classification group across folds. Blue, yellow, and red boxes indicate EEG, EOG, and EMG perturbation, respectively.


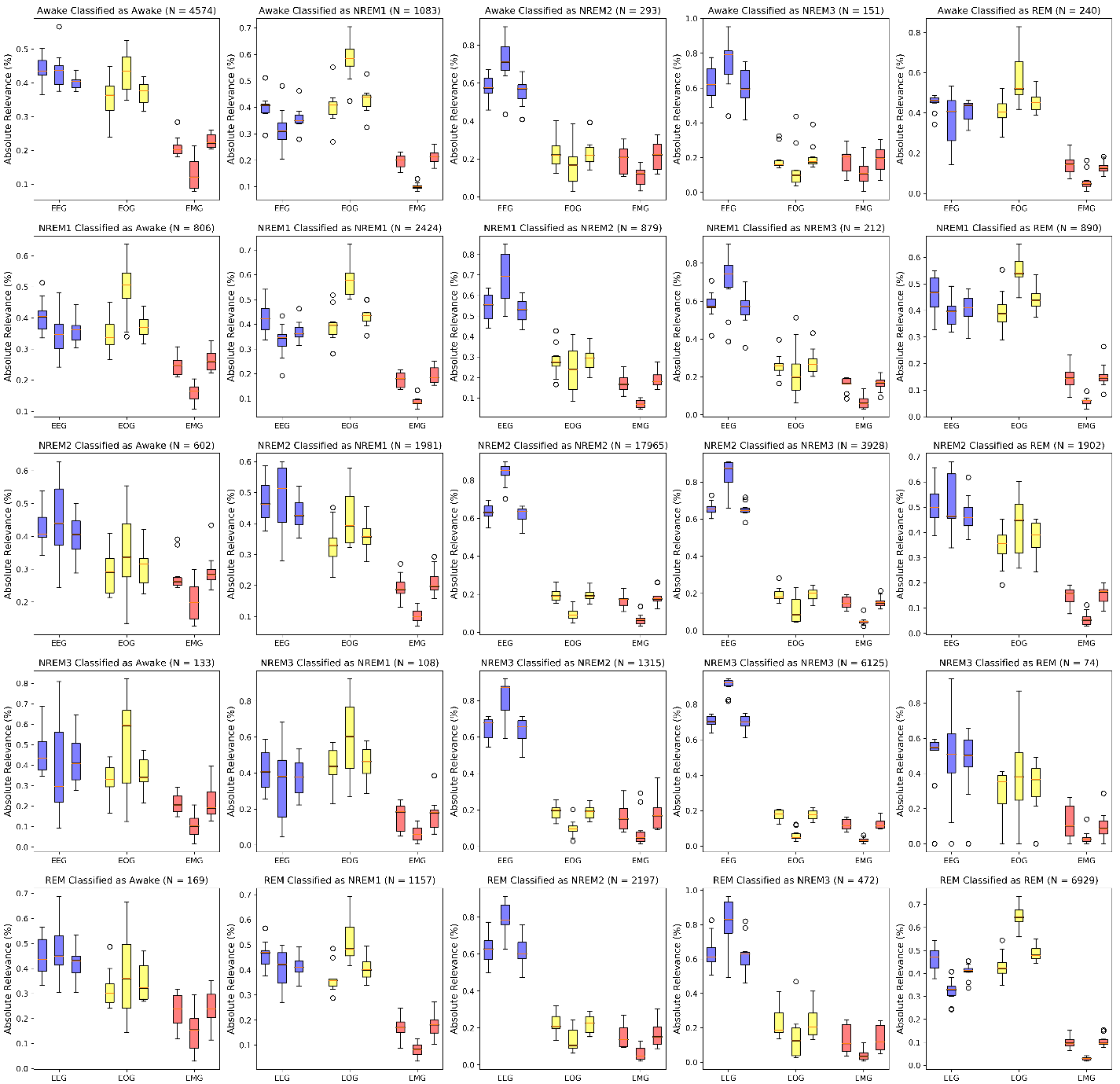


**Supplementary Figure 3.** LRP-based Global Estimation of Modality Importance for All Classification Groups. The boxplots show the LRP explainability results for each classification group across folds. Blue, yellow, and red boxes show the importance of EEG, EOG, and EMG, respectively. From left to right, within each group of three boxes are the relevance results for the LRP ε-rule (0.01), ε-rule (100), and αβ-rule. The number of samples in each classification group is included in the title of each panel. The panels on the left-to-right diagonal show correct classification groups, and off-diagonal panels indicate incorrect classification groups.


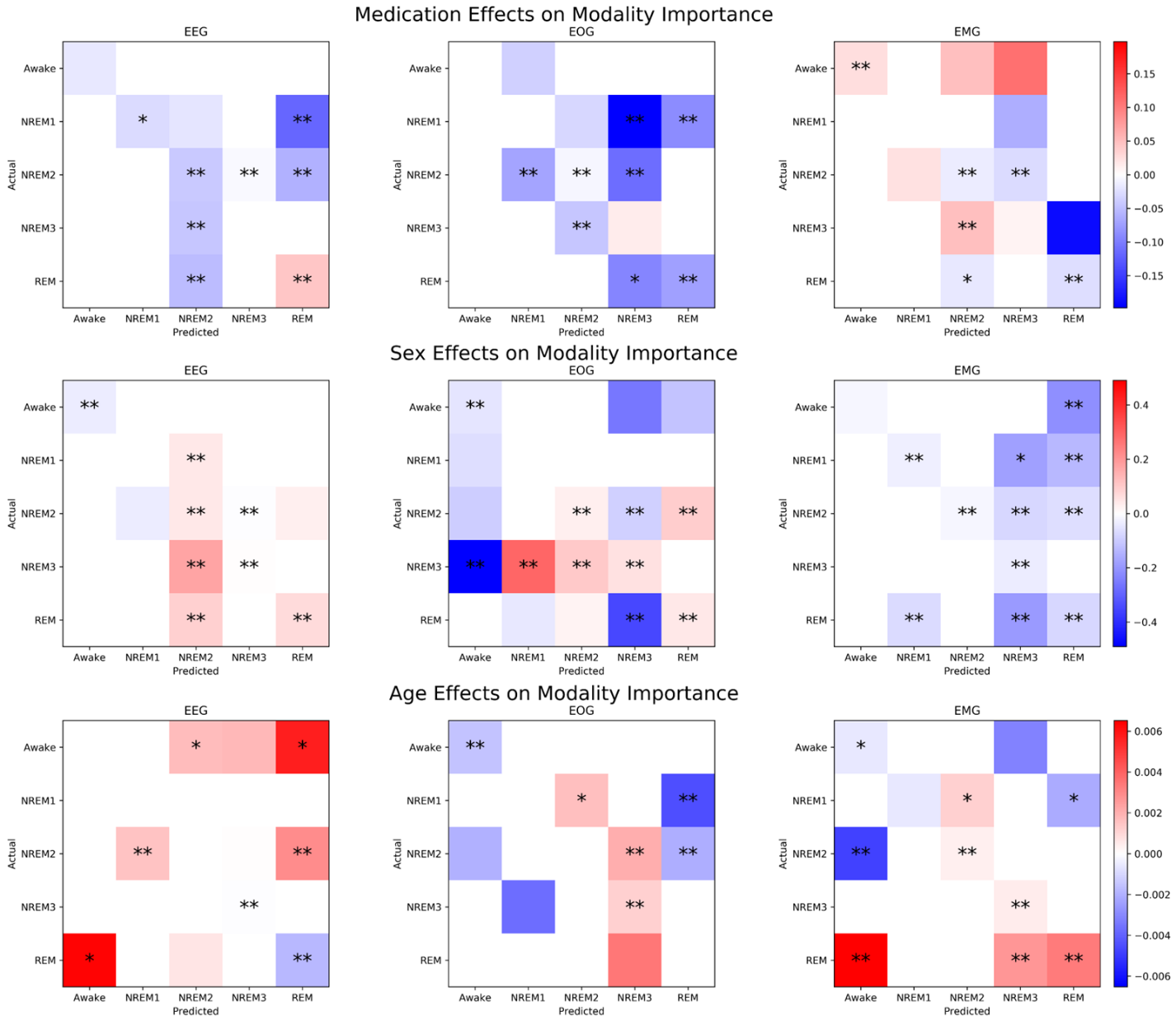


**Supplementary Figure 4.** Effects of Clinical and Demographic Variables upon Local Ablation Modality Importance for All Classification Groups. The top, middle, and bottom rows of panels indicate the effects of medication, sex, and age, respectively, upon the explanations for each modality and classification group. The x-axis of each panel indicates the predicted class, and the y-axis indicates the actual class. The heatmaps indicate the size of the coefficient values resulting from the regression analysis. White squares have insignificant p-values. Squares with color have significant p-values (p<0.05), and squares that have one or two asterisks have p-values of p < 0.01 or p < 0.001, respectively. For medication, a positive coefficient value indicates that temazepam samples had more importance than placebo samples. For subject sex, a positive coefficient value indicates that female samples had more importance than male samples, and for age, a positive coefficient indicates that importance increased with age.


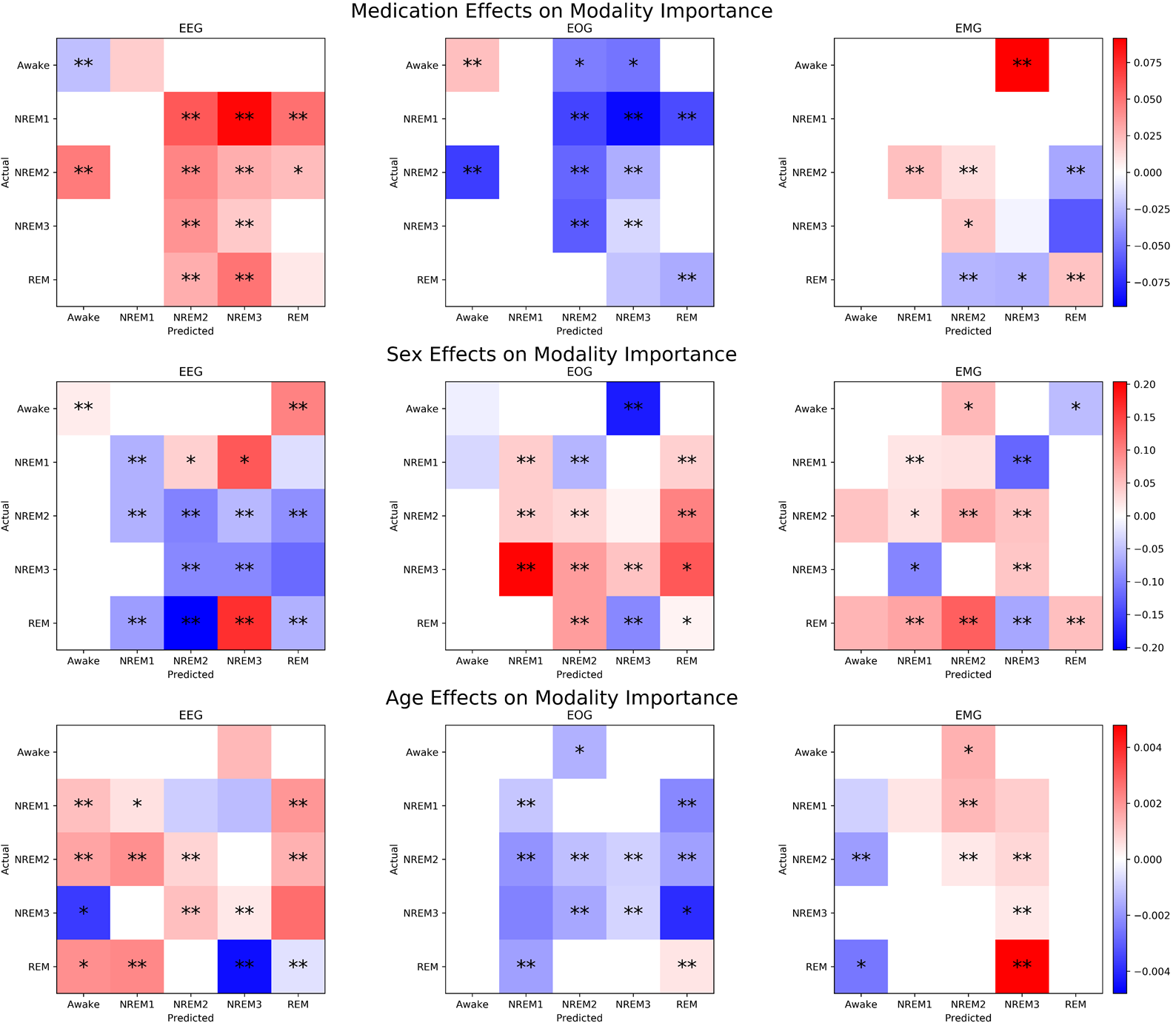


**Supplementary Figure 5.** Effects of Clinical and Demographic Variables upon LRP Relevance for All Classification Groups. The top, middle, and bottom rows of panels indicate the effects of medication, sex, and age, respectively, upon the explanations for each modality and classification group. The x-axis of each panel indicates the predicted class, and the y-axis indicates the actual class. The heatmaps indicate the size of the coefficient values resulting from the regression analysis. White squares have insignificant p-values. Squares with color have significant p-values (p<0.05), and squares that have one or two asterisks have p-values of p < 0.01 or p < 0.001, respectively. For medication, a positive coefficient value indicates that temazepam samples had more relevance than placebo samples. For subject sex, a positive coefficient value indicates that female samples had more relevance than male samples, and for age, a positive coefficient indicates that relevance increased with age.
